# Editorial: Advances in antigen-specific immunotherapies for autoimmune disease management

**DOI:** 10.3389/fimmu.2026.1875877

**Published:** 2026-05-18

**Authors:** Michael A. Firer, Nechama Sharon

**Affiliations:** 1 Adelson School of Medicine, Ariel University, Ariel, Israel; 2 Department Chemical Engineering, Ariel University, Ariel, Israel; 3 Department Hematology, Laniardo Hospital, Netanya, Israel

**Keywords:** autoantibodies, autoimmunity, CAR-T cell, immune tolerance, targeted immunotherapy

Last year, the National Institute of Allergy and Infectious Diseases (NIAID) estimated that about 8% of the US population (~29 million people) suffer from autoimmune diseases (AID) of which there are between 80–150 recognized types. Most of these illnesses are chronic, affect more than one organ, are associated with substantial morbidity and patient care often involves family members. AIDs have emerged as a global health concern. In the US alone, health care costs related to AIDs have reached over $100 billion per year, according to a recent NIH study (1) and AIDs contribute substantially to premature death (2).

The spectrum of AIDs covers almost every organ in the body and while the etiology of these diseases is based on some shared environmental, immunological and genetic factors (3), the devil is in the complex detail of the combinations of these and additional factors, such as gender. Due to this complexity drug therapies for AIDs have, historically, sought to treat common components across the spectrum of disorders. A good example is the use of steroids to control aberrant, self-directed immune responses, a common feature of these diseases. However, the long-term use of steroids is associated with a list of adverse effects related to pan-immunosuppression, despite a drop in recommended doses over recent years (4). And side effects need to be treated, which has led to an increase in polypharmacy that carries its own adverse outcomes (5). Drug therapy for autoimmune diseases is focused on controlling symptoms rather than curing the underlying pathology, exemplified by the use of anti-coagulants in patients with Anti-Phospholipid Syndrome (APS).

AIDs pose a significant challenge in the field of immunology; to decipher the immune system’s failure to distinguish between self and non-self. Despite extensive research, the precise mechanisms that disrupt immune tolerance to self remain elusive, and there is a pressing need for novel therapeutics that can effectively target the underlying causes of AIDs.

Traditionally, AIDs have been ranked as specific or non-specific according to the tissue restriction of their clinical symptoms. Given the significance of the dysfunctional regulation of the immune response in the pathology of these diseases, this system of cataloging is misleading and undermines the complexity of the immune response. On the one hand, AIDs are (often) characterized by the production of a series of autoantibodies, some of whose targets are not disease specific. For example, anti-dsDNA antibodies are a hallmark of Systemic Lupus Erythematosus (SLE) where they form immune complexes and induce nephritis; however, this process also occurs in other AIDs (6). On the other hand, T cell responses are tissue-specific, even in multi-system diseases such as SLE, both at the level of autoreactive T cells (7), and for T regulatory cells (8).

Enter antigen-targeted B-cell and T-cell immunotherapies, which, after decisive and life-saving proofs-of-concept in cancer therapy, are also now being applied to restore immune homeostasis by targeting specific self-antigens implicated in these diseases. However, the development and clinical application of such targeted immunotherapies are still in their nascent stages, necessitating further exploration and validation. Antigen-targeted immunotherapies raise the opportunity to develop more effective and controlled therapies for AIDs rather than deferring to pan-immunotherapies which, while effective may carry long-term safety issues (9, 10).

With these issues in mind, the current Research Topic of Frontiers in Immunology entitled *“Advances in antigen-specific immunotherapies for autoimmune disease management”*, aims to explore the development and application of targeted immunotherapies for autoimmune diseases, with a focus on antigen-specific approaches. The primary objective for selection of the 16 articles in this Research Topic was to investigate how these therapies can be harnessed to restore immune homeostasis and provide more effective treatment options for patients suffering from AIDs. Key questions addressed by the authors include understanding the mechanisms leading to immune dysfunction in particular AIDs, and the efficacy of targeted immunotherapies, both in more common and rare AIDs.

Several approaches have been tested to control the production of pathogenic autoantibodies. The article by Shai et al. reviewed mechanisms of B cell tolerance with the aim of identifying those that may be triggered therapeutically in the treatment of SLE. They suggest several approaches, including pharmacological, that would potentially enhance intrinsic B cell tolerance.

Another arm of the immune response dysfunctional in AIDs is the normally balanced and regulated production of cytokines. Lv et al. reviewed the bidirectional interaction between dendritic cells and T follicular helper cells (TfH). Their work elucidated the pathway by which specific mature Dendritic Cell (DC) subsets in the intestinal inflammatory microenvironment drive TfH cell differentiation to secret cytokines that recruit CCR7+ DCs and CXCR5+ lymphocytes to form structural lymphoid clusters. The DC-TfH cell interactions also enhance Th1/Th17 differentiation and weaken T regulatory capacity, leading to inflammation and tissue damage in inflammatory bowel diseases.

Understanding the molecular machinery responsible for effector immune responses has led to several breakthrough technologies that are impacting patient survival and quality of life. This is particularly the case with the ever expansion in the spectrum of humanized therapeutic monoclonal antibodies. This is described in the papers by Lee and Kahaly and Chen et al, who reviewed the effect targeted immunotherapies in the treatment of patients with Graves’ hyperthyroidism and in the rare AID Generalized pustular psoriasis, respectively.

Another breakthrough technology is the use of Chimeric Antigen Receptor T-cells (CAR-T cell), initially termed “T-bodies” by its inventor Zelig Eshhar (11). Several CAR-T cell therapies have received FDA approval for B-cell hematological cancers (12) and there is keen interest in applying CAR-T cell therapies to AIDs. Both Rangel-Pelaez et al. and Yu et al. reviewed the experience acquired in the application of CD19 targeted CAR-T cells for targeted immunotherapy for several common autoimmune rheumatic diseases such as Rheumatoid Arthritis (RA), and SLE. CD19 targeted CARs would potentially remove all B cell clones. To avoid this situation, Rangel-Pelaez et al. also discussed efforts to develop CAR-T cells incorporating ligands that only bind autoantibody-producing B cells. This approach should be most effective for AIDs involving a limited number of autoreactive B cell clones whose autoantibodies are directly pathogenic, such as in APS or Idiopathic Thrombocytopenia Purpura (ITP), but would be challenging in diseases involving a wide range of autoreactive B cells, such as in RA and SLE. Xu and Su provided a timely update of the clinical landscape of CAR T cell clinical trials for AIDs. Their study demonstrates the linear and rapid growth of trials between 2018-2024 (when the study ended), particularly in Phase I trials, reflecting the growing interest in this field. Nonetheless, the challenges facing CAR T-cell therapies for cancer, such as high cost, side effects (neuropathy, cytokine release syndrome) and effect duration also apply to AIDs. As of January 2025, the FDA has yet to approved CAR T-cell therapy for AIDs.

Several articles reported original research testing novel targeted immunotherapies. Kakabadse et al. engineered mouse CD4+ T regulatory cells to recognize a hybrid insulin peptide in order to attract and suppress islet-specific Cd8+ T cells. When injected into non-obese diabetic mice, these engineered cells reduced insulinitis and trevented spontaneous diabetes.

Based on the successful use of the anti-CD20 monoclonal antibody Rituximab in eliminating disease-related B-cells such as in B-cell leukemia, Li et al. reasoned that this approach would reduce the production of anti-PLA2R antibodies, which are crucial in the development of nephrotic syndrome. The aim of the study was to overcome the dependance on calmodulin inhibitors (CNI) id patients with primary membranous nephropathy (PMN). They found that Rituximab therapy led to complete clinical and immunological remission in PMN patients dependent on or partially responsive to long-term CNI therapy, reducing recurrence and minimizing prolonged immunosuppressive therapy risks.

Returning to the problem of long-term use of corticosteroids mentioned earlier, Shirai et al. tested the feasibility of the drug’s removal from patients with Takayasu arthritis (TA), a rare, chronic large-vessel vasculitis that causes inflammation, stenosis, and aneurysms in the aorta and its main branches. Patients with TA were treated with Tocilizumab, a monoclonal antibody to the IL-6 receptor, while tapering off prednisone, resulting in 44.4% of the patients achieving corticosteroid withdrawal. This study highlights the potential of replacing a non-specific pan-immunosupressive drug with a immunotherapy directed at disease pathology.

Wang et al. are also developing a therapeutic strategy that targets the immune mechanism responsible for restoring tolerance towards the specific self-antigens. They developed a platform in which nanoparticles coated with self-antigen-derived peptides are delivered to liver sinusoidal endothelial cells (LSECs). These cells are known for their role in inducing antigen-specific T-cell tolerance. The authors demonstrate the potential of the strategy in several animal models of AID.

These studies, along with case reports by Sun et al, Yang et al, Du et al, and Song et al, showcase the potential of replacing many of the current symptom-based therapies for patients with AIDS with those that focus on the cellular mechanisms that lead to the breaking of immune tolerance to specific self-antigens. We are still in the early days of this transformation. While some of these approaches are progressing through clinical evaluation, basic research must continue to focus of the basic immunology of autoimmunity in collaboration with biotechnology to develop applications that are translatable and will lead to improved quality of life for patients.

In conclusion, we would like to thank to Editorial team of Frontiers in Immunology for the opportunity to edit this Research Topic, and in their helpful and professional processing of the articles.
